# Shedding Light on the Molecular Recognition of Sub-Kilodalton Macrocyclic Peptides on Thrombin by Supervised Molecular Dynamics

**DOI:** 10.3389/fmolb.2021.707661

**Published:** 2021-08-31

**Authors:** Mahdi Hassankalhori, Giovanni Bolcato, Maicol Bissaro, Mattia Sturlese, Stefano Moro

**Affiliations:** Molecular Modeling Section (MMS), Department of Pharmaceutical and Pharmacological Sciences, University of Padova, Padova, Italy

**Keywords:** molecular recognition, macrocyclic, supervised molecular dynamics, thrombin, tetrapeptide

## Abstract

Macrocycles are attractive structures for drug development due to their favorable structural features, potential in binding to targets with flat featureless surfaces, and their ability to disrupt protein–protein interactions. Moreover, large novel highly diverse libraries of low-molecular-weight macrocycles with therapeutically favorable characteristics have been recently established. Considering the mentioned facts, having a validated, fast, and accurate computational protocol for studying the molecular recognition and binding mode of this interesting new class of macrocyclic peptides deemed to be helpful as well as insightful in the quest of accelerating drug discovery. To that end, the ability of the in-house supervised molecular dynamics protocol called SuMD in the reproduction of the X-ray crystallography final binding state of a macrocyclic non-canonical tetrapeptide—from a novel library of 8,988 sub-kilodalton macrocyclic peptides—in the thrombin active site was successfully validated. A comparable binding mode with the minimum root-mean-square deviation (RMSD) of 1.4 Å at simulation time point 71.6 ns was achieved. This method validation study extended the application domain of the SuMD sampling method for computationally cheap, fast but accurate, and insightful macrocycle–protein molecular recognition studies.

## Introduction

The ever-increasing expeditious development of computer hardware, software, and algorithms has positively contributed to many domains of research such as drug design. The developed computational methods, namely, molecular docking and molecular dynamics (MD) simulations, to name but two, greatly reduce the time and cost of drug development, in a way that *in silico* modeling tools are highly utilized in the research ambit of drug discovery (Muegge et al., 2017; Salmaso et al., 2017; Lin & Li, 2020). Particularly, the investigation of the binding mode, following the steps of varied ligand–target recognition pathways, and exploring their interactions have been claimed to be the area of impressive application of MD computational protocols (Salmaso et al., 2017).

Molecular dynamics simulations are considered an endorsed computational method in which by integrating the numerical solution of the Newton equation of motion, the time-dependent evolution of a molecular system can be revealed and described. However, obtaining a complete molecular recognition trajectory leading to binding, from the unbound to the bound state, is a rare event, and to capture moments of importance, therapeutically speaking, *via* a free classical molecular dynamics approach requires a long microsecond timescale and therefore massive computing resources even with the novel GPU-based protocols (Buch et al., 2011; Dror et al., 2011; Shan et al., 2011).

Our in-house alternative MD approach, compared to the classical method, named supervised molecular dynamics (SuMD), improves the efficiency of sampling a binding event and decreases the simulation time from a microsecond (µs) to a nanosecond (ns) timescale (Sabbadin and Moro, 2014). To do that, it applies a tabu-like algorithm to monitor the distance between the ligand center of mass and the target binding site center of mass during a short classical MD simulation; only productive simulations in terms of reducing this distance are considered productive. Despite the exploration of the recognition event, SuMD has been previously proved to be able to reproduce the experimental bound state of various kinds of complexes with great geometric accuracy. Its already validated application domain covers the molecular recognition simulation of small molecules, natural linear peptides, most classic peptidomimetics, and nucleic acids (Bissaro et al., 2020).

Among different classes of compounds, macrocycles are attractive structures for drug development, due to their potential in binding to “undruggable and canonical small molecules or proteins” (Kale et al., 2019). Macrocyclic peptides represent an efflorescing class of molecules potentially targeting numerous disease-related protein targets otherwise intractable *via* established pharmacological approaches (Passioura, 2020). Several remarkable characteristics can be considered for this class of molecules. First, compared to linear peptides, they are relatively stable and less prone to protease degradation. The cyclization also confers advantages such as having a compromised state between a flexible and a preorganized structure required for dynamic interactions with protein targets with a conformational bias; a reduced binding entropy cost can be imagined compared to their linear counterparts (Giordanetto and Kihlberg, 2014). However, it is worth to mention that due to the reduced accessible conformational states, shifting the structure—upon macrocyclization—toward states that can anticipate bioactivity for a specific target binding site is consequential because otherwise the non-bioactive conformation stabilization can slow down the binding. Therefore, identification of highly populated conformations of macrocycles is of significance when it comes to drug design (Kamenik et al., 2018). Moreover, it has been shown that macrocyclic peptides are capable of selectively binding to relatively shallow, flat, and featureless protein surfaces often involved in clinically important protein−protein interactions (PPIs), in a fashion similar to antibody-based therapeutics and conversely to small molecules which generally need a pocket to bind (Deyle et al., 2017; Vinogradov et al., 2019). Furthermore, thanks to their amino acid composition, a low innate toxicity is anticipated which is of advantage as therapeutic modalities. Being synthetically accessible makes possible lead optimization attempts and altering biophysical properties in terms of binding affinity and specificity, proteolytic stability, and/or solubility improvement for a particular purpose. A variety of macrocyclization reactions have been devised over the years, and now different topologies can be easily synthetically available (Vinogradov et al., 2019). However, this interesting class of molecules has been underrepresented in numbers and diversity in the available libraries (Kale et al., 2019). In recent years, innovative approaches evolved for further development of cyclic peptides, such as generating and screening large combinatorial cyclic peptide libraries using *in vitro* display. These attempts have increased the availability and potential screening of ten to hundreds of thousands up to 1 trillion compounds or more highly diverse macrocycles with extraordinary target affinity, selectivity, and bioactivity (Deyle et al., 2017; Taylor et al., 2017; Kale et al., 2019; Passioura, 2020). In a recent research project of Kale et al., *via* novel thiol-to-amine cyclization reactions, they introduced a strategy that enables the generation of a high-yield purification-free large library of diverse macrocycles to screen for various targets in an efficient, relatively small-effort manner. Generating a library containing 8,988 macrocycles of sub-kilodalton molecular weight (ideal for addressing the lingering challenge of macrocycles) and screening of this library against thrombin and other homologous targets identified a potent selective thrombin inhibitor called P2 (Ki = 42 ± 5 nM) (Kale et al., 2019).

Given the emerged perspective stemming from all referred above, having a reasonably fast and accurate computational method like SuMD for studying the molecular recognition pathway and reproduction of an experimentally comparable binding mode of this promising macrocyclic class of peptides is deemed significant. With that intent, through this study, the ability of the SuMD protocol in the reproduction of the X-ray crystallography final bound state of the candidate macrocyclic peptide P2 as a potent thrombin inhibitor was evaluated.

**FIGURE 1:**
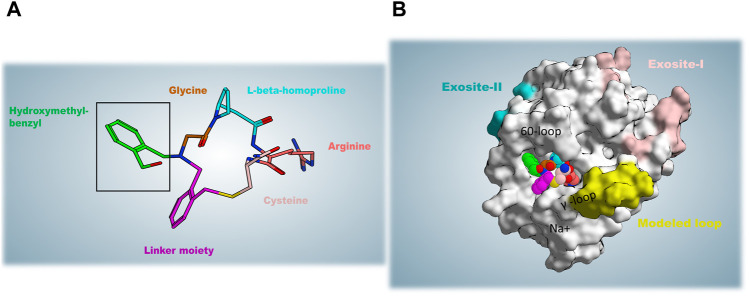
**(A)** The structure of P2 is shown; the hydroxymethyl-benzyl moiety (in green) that is lacking in the P1 macrocycle is highlighted by a black frame. **(B)** Thrombin in complex with P2; thrombin structural determinants for its function and client recognition are also reported.

P2 is a tetrapeptide composed of “glycine”–“L-beta-homoproline”–“ arginine”–“cysteine” cyclized with a linker of di-bromomethyl benzyl and an N-(2-(hydroxymethyl)benzyl) substituent coming from an additional reaction of the linker (Figure 1). P2 is proved to be a highly selective inhibitor for thrombin with a snug fit of the specific backbone to the target, while it did not show any considerable inhibition for other homologous structurally and functionally similar proteases such as activated protein C (APC) and tissue plasminogen activator (tPA), to name but two (Kale et al., 2019). A representation of thrombin in complex with the P2 structure is shown in Figure 1. During library screening, another macrocycle called P1 with a similar structure to P2 and merely lacking the hydroxymethyl-benzyl moiety showed three orders of magnitude lower inhibition constant than P2 (Kale et al., 2019). Given that and the fact of any experimentally reported binding state not being available for P1, the idea to try simulating a probable binding mode of P1 in addition and possibly hypothesizing the inhibition potency difference through our *in silico* studies was emerged.

The protein target in this study, thrombin, is a typical trypsin-like serine protease and the final generated protease during the blood coagulation cascade. It is worth raising the point that distinct structural features are present in this single protease for the recognition ability of different substrates in a specific manner (Huntington, 2005; Huntington, 2008). As reported in Figure 1, the walls of a deep active site cleft—often referred to as canyon—are formed by the two insertion loops known as the 60-loop and γ-loop. The upper 60-loop, is a rigid, hydrophobic cap over the active site, while the more hydrophilic and flexible γ-loop is situated at the downside of the cleft. A constricted access to the catalytic site of thrombin is provided only to proteins with long, flexible substrate loops (Huntington, 2005). The substrate recognition within the active site of thrombin occurs thanks to favorable interactions between the P1 residue [according to the Schechter and Berger nomenclature of amino acid residues around the substrate scissile bond (Schechter and Berger, 1967)] and the deep acidic S1 pocket (Asp189, Ser190, Gly219), as well as the presence of hydrophobic/aromatic residues N-terminal to P1 occupying the S2 pocket (Tyr60A and Trp60D as the main residues) and S3 (the aryl-binding pocket composed of Trp215, Leu99, Ile174) (Huntington, 2005; He et al., 2015; Fong, 2017). Apart from the active site, three other regions are involved in the diverse specific recognition of different substrates. There are two electropositive exosites, termed anion-binding exosites (ABEs) and a sodium-binding site. The all-natural thrombin substrate directly or *via* cofactor mediation establishes contacts with at least one exosite and usually both; this represents the prerequisite event to form initial stable complex conformation needed for the peptide bond cleavage (Chahal et al., 2015; Huntington, 2005, 2008). The sodium-binding site, 15 Å away from the catalytic triad (His57, Asp102, Ser195), with Na^+^ coordinated to the main chain oxygen atoms of Arg221a and Lys224 and four conserved water molecules, is considered another allosteric activity modulator site of this protease, helping the maintenance of the hemostatic balance. Upon binding to sodium, thrombin shifts toward a conformation known as “fast conformation” able to cleave all procoagulant substrates such as fibrinogen- and protease-activated receptors more readily. On the contrary, in the Na-unbound “slow” state, the protein C anticoagulant pathway is preferentially activated. Under physiologic conditions, the 140 mmol/L Na^+^ concentration in the blood would not saturate the site, and a present 2:3 ratio of slow: fast states accounts for optimal allosteric regulation of anticoagulant: procoagulant activities and hemostasis (Di Cera, 2003; Huntington, 2008; Kahler et al., 2020).

## Materials and Methods

### Computational Study Infrastructure

This project was carried out on a hybrid GPU–CPU Linux cluster of 280 CPU cores and 30 NVIDIA graphic cards.

### Structure Preparation

To begin with the simulation, the three-dimensional coordinates of the crystal structure of thrombin bound to the P2 macrocycle (PDB ID: 6GWE) were retrieved from the RCSB Protein Data Bank (PDB) with a resolution of 2.3 Å (Kale et al., 2019). Then, using MOE suite (*Molecular Operating Environment (MOE)* & Chemical Computing Group ULC) version 2019.01, the structure was checked and modeled (*via* the loop modeler plugin) for the missing loop, 3D protonated, and energy minimized regarding the energy of the added hydrogens and their positions. For this study, one of each unique chain which is chain A with 257 residues and chain B with 30 residues in their sequence, in addition to the sodium ion bound to the chain A sodium binding loop, was kept. The modeled eight-residue missing loop between Glu146 and Gly150 amino acid sequences comprised of TWTANVGK.

### Solvated System Setup and Equilibration

All MD simulations were carried out using AMBERTools14 (Case et al., 2014). To parameterize the ligand, the *Antechamber* tool (Wang et al., 2006) in conjunction with a general Amber force field (GAFF) (Wang et al., 2004) was utilized to classify atom and bond types, assign charges, and estimate force field parameters. The charge method AM1-BCC of the GAFF which is semi-empirical was used in this study. The solvation box with charge neutrality and physiological ionic strength (0.154 M in Na^+^ and Cl^−^ ions), as well as complex system parameters and topology files, was prepared using tLEaP (Case et al., 2005). Protein and water were represented by Amber *ff14SB* (Maier et al., 2015) and TIP3P (Jorgensen et al., 1983) models, respectively, in the prepared system. In all SuMD replicas, simulation starts with the ligand located 40 Å far from the orthosteric active site at time zero, which is a distance bigger than the electrostatic cut-off term used in the simulation (9 Å with the Amber force field), to avoid premature interaction during the initial phases of SuMD simulations.

All simulation systems were energy minimized through two equilibration steps. Considering 2 fs as a time step equal to the vibrational frequency of bonds, 500,000 steps (1 ns) of NVT in addition to 500,000 steps (1 ns) of NPT simulations were carried out. Gradual reduction of harmonic positional constraints by a force constant of 5 kcal mol^−1^ Å^−2^ was applied in both steps. In the first equilibration, ions (except bounded Na^+^ in the sodium-binding loop) and water were kept free, while protein and ligand atoms were constrained. However, in the second equilibration, the constraints were kept only on the alpha carbons of the protein, as well as ligand atoms and the loop sodium. In both steps, the temperature was maintained at 310 K by a Langevin thermostat with low damping of 0.1 ps^−1^, and in the second NPT step, the pressure was maintained at 1 atm by a Berendsen barostat as well (Berendsen et al., 1984). To calculate electrostatic interactions with a cubic spline interpolation and a 9.0 Å cut-off for Lennard–Jones interactions, the particle-mesh Ewald (PME) method was utilized (Essmann et al., 1995).

### Supervised Molecular Dynamics Production

The SuMD simulations were done in NVT conditions with the temperature equal to 310 K, while the pressure of the system was free to change. To perform a supervised MD simulation, the topology and coordinates of the last frame of the second equilibration phase were used as the starting point. In the configuration file of SuMD, three selected amino acid residues Glu97A, Gly219, Cys191 whose center of mass (CM) approximately defines the binding site CM were inputted. SuMD applies a dynamic selection on the indicated residue position to calculate the center of mass of the binding site. MOE suite was used to determine the center of mass of the co-crystallized ligand regarded as the center of mass of the thrombin active site to be then visually selecting a combination of residues that their center of mass could represent the approximate position of the binding site guiding the supervision. Each SuMD replica was produced on a graphics machine using ACEMD3 (Harvey et al., 2009) as the MD engine. The length of the SuMD steps for SuMD replicas was set to either a 600 ps or a 1 ns time window.

### Free (Unsupervised) Classical Molecular Dynamics Production

For each cMD, after system preparation and equilibration steps, the ACEMD3 (Harvey et al., 2009) engine was used with the same settings, except for the simulation length, of the cMD simulation in each SuMD step.

### Visualization of the Molecular Dynamics Trajectories

Visual Molecular Dynamics (VMD) (Humphrey et al., 1996) and MOE suite (*Molecular Operating Environment (MOE)* & Chemical Computing Group ULC) were utilized during this project for molecular visualization and analysis of the trajectories.

### Trajectory Versus Trajectory Root-Mean-Square Deviation Calculation

**FIGURE 2:**
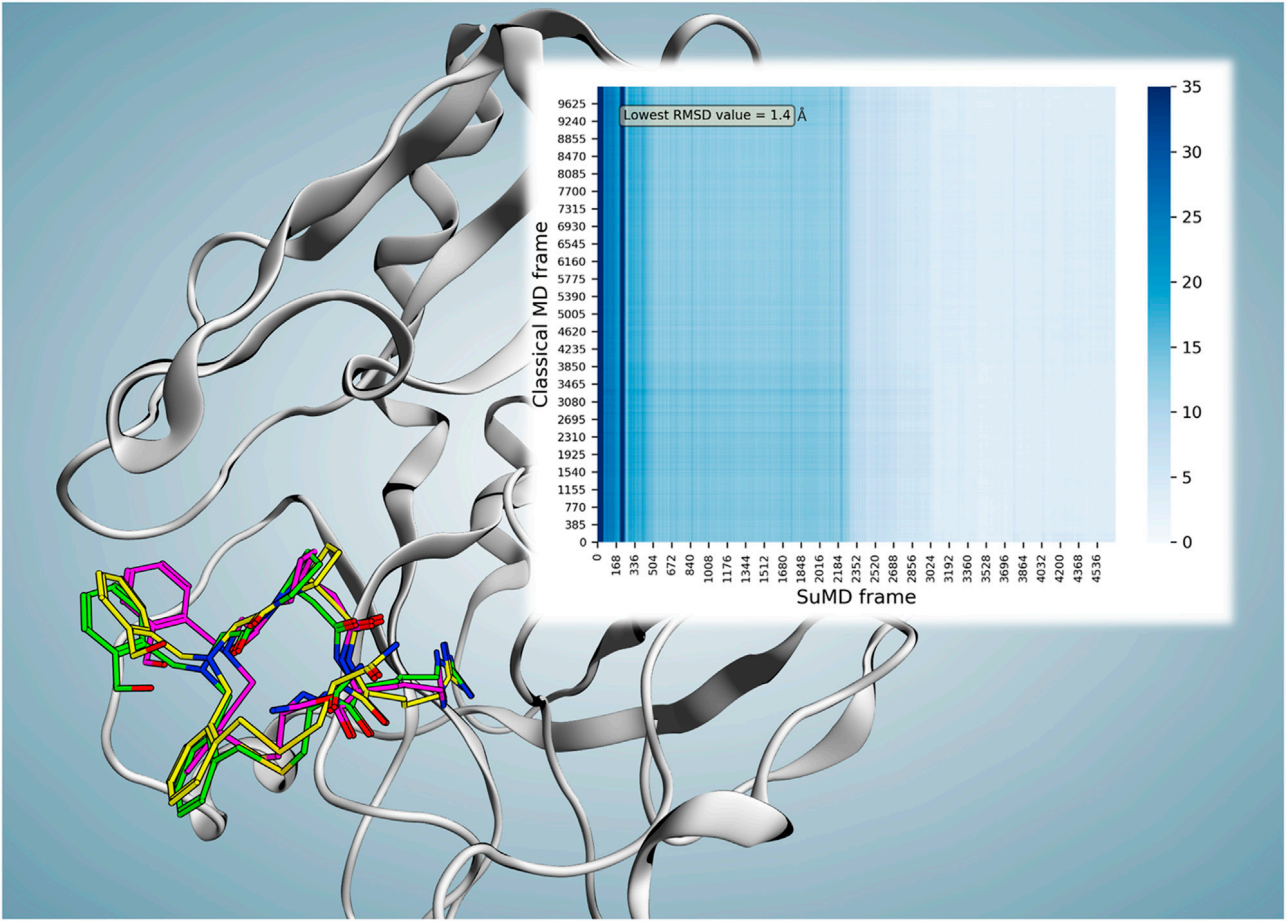
Superposition of the P2-reported X-ray crystallography conformation (magenta), frame number 102 (2.04 ns) of reference cMD (green), and frame number 3,579 (71.56 ns) of replica 74 (yellow; the frame with the lowest RMSD value compared to both reported binding conformation and the parallel trajectory analysis reference resulted in the frame). The minimum obtained RMSD compared to the reported binding conformation showed a value of 2.27 Å at time point 71.56 ns. However, considering the observed instability of the reported conformation, parallel frame RMSD calculation of the replica 74 trajectory versus reference cMD trajectory was performed which resulted in the minimum RMSD value of 1.4 Å at 71.56 ns for frame number 3,579 (the same frame with the lowest RMSD value compared to the reported binding conformation). The heat map of the parallel trajectory RMSD analysis of replica 74 versus reference cMD is shown on the top right.

Using MDAnalysis (Michaud-Agrawal et al., 2011; Gowers et al., 2016), a matrix of frames related to the cMD of reference against frames of each SuMD replica was set for comparative root-mean-square deviation (RMSD) calculation. Then *via* the Seaborn Python library (Waskom et al., 2020), a heat map of the resulting RMSD calculation was illustrated (Figure 2).

### MM-GBSA Energetic Profile Analysis and Clustering

All total free energy calculations in this work were computed using the MMPBSA.py tool (Miller et al., 2012) using the GB-OBC(II) Born solvation model and no entropy calculation. To identify other energetically favorable binding sites and elucidate a P2 ligand−protein recognition scenario, the trajectories of 99 SuMD replicas of P2 were first solvent-dried, aligned, merged, and ten times strided as input for positional clusterization. To do so, ligand sets of coordinates (each set of coordinates corresponds to the ligand conformation in a frame) after discarding noise sets considering a cosine similarity value of 0.01 were clusterized using the OPTICS algorithm of Scikit-learn (Pedregosa et al., 2011). Thereafter, given the MM-GBSA value of the included ligand coordinates in each cluster, the representative ligand conformation with the most favorable energetic value was selected for the corresponding cluster.

## Results

### Study Principal Outcomes

This study was conducted aiming to extend the application domain of our molecular dynamics supervision method for studies related to models of the sub-kilodalton macrocyclic peptide–protein binding event. As a case study, the SuMD ability to reproduce the X-ray crystallography bound state of the P2 macrocyclic peptide to thrombin was evaluated. To that end, 99 SuMD simulations were performed starting from an unbound state obtained by separating P2 from its binding site by around 40 Å. Among 99 SuMD replicas, 84 trajectories finished with the ligand arriving in the proximity of the binding site and its sub-pockets with different binding orientations and conformations, while 15 trajectories ended with the ligand stopping over a varied site categorized as “failed” based on SuMD termination criteria (far from the binding site). Overall, five trajectories concluded with the ligand reaching the narrow S1 pocket (guanidinium moiety entering S1), all below 100 ns of SuMD-productive simulation time.

To better compare the SuMD results with the experimental structure, the X-ray crystallography complex (reference) was subjected to 200 ns of cMD, allowing to have both systems in similar conditions: equilibrated and relaxed in a fully explicit solvent environment. In fact, during the initial 4 ns of the cMD, a fluctuation within the range of experimental resolution (2.3 Å) was observed, while after 4 ns, a more significant shift of the macrocycle occurred as confirmed by a drop of its RMSD values to above 3 Å and below 5.66 Å until the end (200 ns) was detected (Supplementary Video S1). The mean calculated RMSD during this trajectory was 3.57 ± 0.47 Å (Supplementary Figure S1). Those RMSD values highlight a discrepancy between the experimental bound conformation and the one assumed once the system is equilibrated in a fully explicit solvent suggesting that the cMD could represent a more adequate comparison for SuMD. Indeed, we performed a frame-to-frame analysis of the SuMD trajectory versus cMD trajectory monitoring the ligand RMSD. Among all replicas, replica 74 is deemed the best-produced binding event trajectory for P2. This SuMD simulation (replica 74) with 94 ns duration reproduced a possible binding event trajectory with the most comparability to the X-ray crystallography binding mode. The minimum obtained RMSD compared to the X-ray conformation showed a value of 2.27 Å at time point 71.56 ns. However, considering the cMD trajectory as a reference, the minimum RMSD value is 1.4 Å at the same time point (71.56 ns, frame number 3,579) versus frame number 102 (2.04 ns) of reference cMD (Figure 2).

**FIGURE 3:**
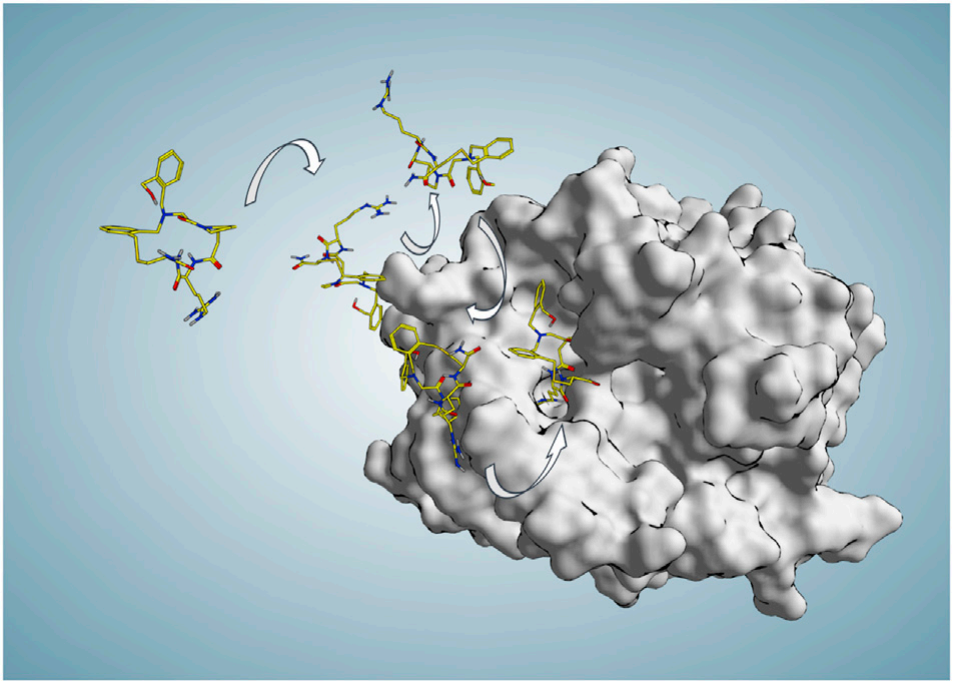
Some representative P2 poses along the binding trajectory (94 ns) produced in SuMD replica 74 during which P2 starts approaching the active site from 30 Å far from any protein atom at time zero and reaches the binding site and S1 pocket in an experimentally comparable binding mode (RMSD_min_ = 1.4 Å at 71.56 ns).

**FIGURE 4:**
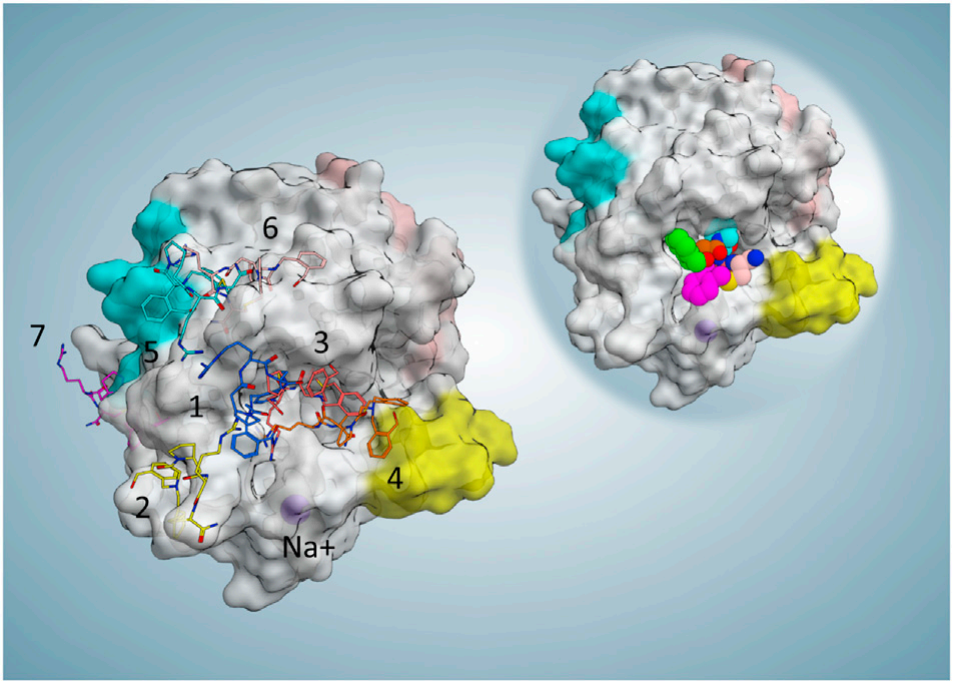
Representative frame (minimum MM-GBSA free binding energy in each cluster) and position of each cluster. For the collective illustration of all representing poses on one protein surface, the molecular surface of the reference PDB is selected to be shown here. On the top right reference, the complex is shown to indicate the active site position.

The simulation started with P2 located 40 Å far from the orthosteric active site (AS) at time zero (Supplementary Video S2), and then upon approaching the AS, the first stable binding occurred from time point 5.5 ns until 43 ns with a mean MM-GBSA free energy (∆G) of −27.1 kcal/mol. As this stopover had enough residence time to break the progressive and continual approach of the ligand, it can be defined as a meta-stable binding site. Afterward, for around 4 ns from time point 48 ns, another stable contact near the active site (∆G = −18.9 kcal/mol) was seen as the ligand was transitioning to the active site area. Then, from 54 ns, a favorable orientation of P2 facilitated the entrance of the fundamental guanidinium moiety to the S1 pocket. From that point, an initial evolution of the final binding state phase was followed by fluctuating but stable similar conformations until the end. The mean total ∆G of the last 22.44 ns (from the RMSD_min_ frame until the end) resulted in a value of −27.3 kcal/mol compared to the calculated mean total ∆G value of −32.2 kcal/mol in the same duration (22.44 ns) of reference cMD, showing a similar with no meaningful MM-GBSA difference (∆∆G < 5 kcal/mol) energetic profile. Some of the most relevant ligand P2 conformations during this binding trajectory are presented in Figure 3. To evaluate the P2 flexibility, we calculated its RMSD during the best five trajectories; the ligand fluctuates until 5.7 Å, suggesting that a certain flexibility is explored during the recognition (Supplementary Figure S2). To identify significant states during the P2 recognition and their corresponding meta-stable binding sites, all the conformations sampled during all the SuMD trajectories were geometrically clustered resulting in seven clusters (Figure 4). All the clusters showed a favorable average of MM-GBSA binding free energy values compared to the calculated value for the reference binding conformation in the canonical binding site (active site) (Table 1). This outcome suggests multiple energetically favorable binding patches on the thrombin surface for P2. Among all the clusters, the seventh cluster comprised of 2–3 times higher number of frames with the minimum average free energy value of −33 kcal/mol. The position of this cluster population was identified near exosite II. Given high population of the seventh cluster, a highly favorable binding free energy value—closely comparable to the canonical binding site—of this positional cluster is near exosite II which is considered an important contact point for natural thrombin substrates to form an initial stable complex conformation required for the peptide bond cleavage; it can be hypothesized that thrombin inhibition by P2 might be resulted from dual-site inhibition, i.e., allosterically preventing the selective stable recognition of substrates in addition to occupying the orthosteric proteolysis site and thus being a potent thrombin inhibitor.

**TABLE 1:** Size and energetic analysis of all the clusters obtained during P2 SuMD simulations.

Cluster No.	Number of included frames	Average MM-GBSA total ∆G (kcal/mol) (rounded)
1	459	−22
2	309	−28
3	460	−27
4	356	−32
5	438	−26
6	275	−28
7	890	−33

Several conformations showed a favorable MM-GBSA binding free energy value suggesting multiple energetically favorable binding states on the thrombin surface for P2.

### Elucidation of the Role of Hydroxymethyl-Benzyl Moiety

**FIGURE 5:**
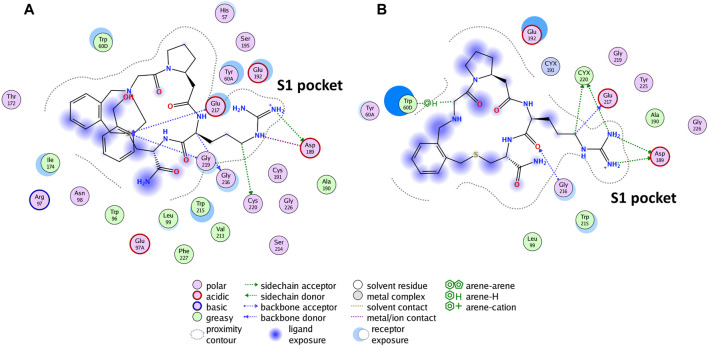
The reported binding mode interactions of P2 **(A)** and the interaction panel of P1 simulated the final stable conformation **(B)**.

Asp1 shares the same structure of P2 except for the presence of a hydroxymethyl-benzyl structure on the latter; a similar binding mode and orientation of the ligand with the guanidinium moiety entering the S1 pocket and the macrocycle occupying the rest of the active site could be hypothesized. For further witnessing of SuMD helpful implication in depicting the molecular basis of the recognition of this class of compounds, we investigate the hydroxymethyl-benzyl role that leads to an increased inhibition activity (three orders of magnitude); SuMD simulations for P1 were additionally performed until reaching a representative replica (Supplementary Video S3) in which P1 fully enters the active site S1 pocket to establish a salt bridge with Asp189. After 18 replicas (in 16 replicas, P1 reached the binding site in different final binding modes, among which six replicas had the supposed orientation and one had expected orientation while entering the S1 pocket), we obtained a possible binding trajectory in which during 34.84 ns of simulation, P1 reached the active site with guanidinium partly inside the S1 pocket as supposed. For further evolution and reaching the most stable conformation, the simulation continued with 50 ns of cMD. After that, a stable conformation was achieved, having a salt bridge with Asp189 and contacting four of the same reference P2 interacting residues (Asp189, Cys220, Gly216, Glu217) (Figure 5).

**FIGURE 6:**
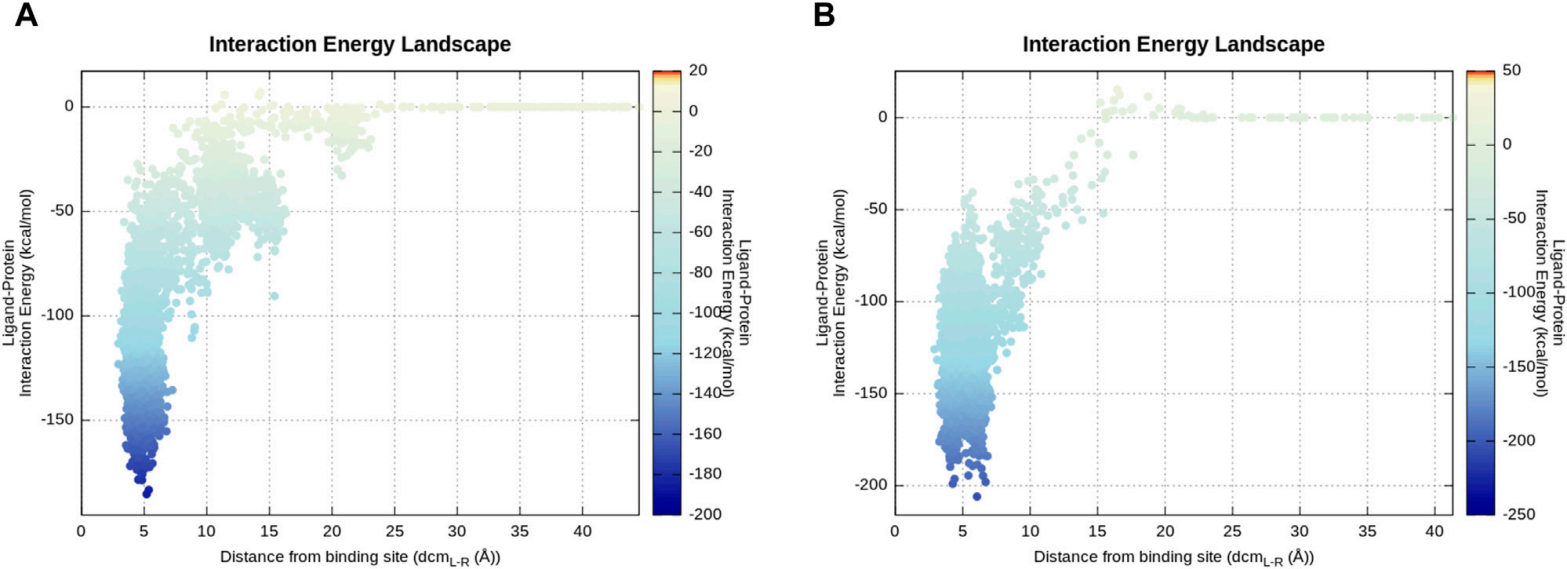
Energy landscape of P2 **(A)** and P1 **(B)** representative produced trajectories. The interaction energy calculation is based on the *mdenergy* function of VMD (Humphrey et al., 1996) and plotted *via* an in-house pepSuMD analyzer (Salmaso et al., 2017). **(A)** Along this trajectory, 2–3 local minima can be seen which correspond to meta-stable binding sites for P2. **(B)** P1 directly goes to the canonical active site during this representative trajectory.

To compare P1 and P2 from an energetic point of view, the mean total MM-GBSA binding free energy during 50 ns of cMD trajectories was taken into consideration. The calculated total ∆G _P1_ = −20.2 kcal/mol and total ∆G _P2_ = −29.3 kcal/mol show a more favorable energy profile in P2 as expected. To be confident about the compared value correctly associated with the final evolved stable P1 binding conformation, similar total ∆G _P1_ = −20.37 kcal/mol was obtained for the last 6 ns of the P1 continued cMD (RMSD_last 6 ns_ = 1.4 ± 0.4 Å). The energy landscape of P1 and P2 trajectories (Figure 6) indicates a similar profile characterized by a large number of energetically stable frames when the distance between the centers of mass (dcm_L-R_) is in the range of 3–7.5 Å. This observation suggests that many ligand states, even if they present different binding modes, contribute to a stable protein–ligand association. The presence of metastable binding sites far from the active site (dcm_L-R_ > 10 Å) is slightly more pronounced in the representative trajectory of P2 where three transient spikes are evident at dcm_L-R_ 9, 15, and 20 Å.

Additionally, to compare P1 and P2 structural characteristics and their possible effects on each ligand dynamics and binding during the SuMD condition, the representative replica of P2 and the P1 residue were selected for further analysis. Given the experimental final binding state of P2, an internal hydrogen bond (2.15 Å/H-O) between the hydrogen atom of the hydroxyl group of the hydroxymethyl-benzyl moiety and the nearby carbonyl group of the macrocycle ring can be seen. This hydrogen bond during the produced binding event trajectory (replica 74) sustains an average value of 2.72 Å (H-O). Considering that, it could be hypothesized that this present internal bond thanks to the hydroxymethyl-benzyl moiety which is absent in P1 contributes to a less flexible structure and a biased maintained conformation necessary for the observed favorable snug-fit binding. To corroborate this idea, the average RMSD of the mutual macrocycle ring of P2 and P1 during the time, in addition to the RMSD of the whole structure of each ligand along their representative SuMD trajectory, was calculated. For this RMSD calculation, all frames of each representative replica were aligned on the comprising atoms of the mutual ring of the corresponding replica’s first frame separately. The calculations obtained in this way indicate the flexibility of the mutual ring and each ligand and not the ligand transition during their molecular dynamics trajectory. The achieved values of the mutual ring and the whole ligand in the P2 trajectory were, respectively, four times and 2.7 times less than calculated values for P1 (average RMSD_ring/P2_ = 0.41 ± 0.15 Å, average RMSD _ligand/P2_ = 1.62 ± 0.4 Å; average RMSD _ring/P1_ = 1.68 ± 0.22 Å, average RMSD _ligand/P1_ = 4.43 ± 0.56 Å). Thus, as expected, this result can quantitatively show a more biased stable conformation for P2 during time compared to P1.

## Discussion

In this study, the ability of the SuMD protocol in the reproduction of the X-ray crystallography final binding state of the candidate macrocyclic tetrapeptide P2—from a novel library of 8,988 sub-kilodalton macrocyclic peptides—bound to thrombin to inhibit its activity was successfully investigated (minimum RMSD of 1.4 Å at 71.56 ns). The outcomes reported that more than 80 percent of trajectories reached the canonical binding surface in varied conformations below or around a hundred nanoseconds, and near five percent mimic the experimentally solved final bound state for this class of macrocyclic peptides to a challenging target, characterized by a narrow active site cleft and deep significant-for-activity sub-pocket (S1). These results reiterated and extended SuMD high value as a computational protocol to explore the recognition pathway. Additionally, based on the observations, SuMD can be regarded as an insightful tool in terms of meta-stable binding site identification, as well as the binding mode and molecular recognition pattern elucidation of sub-kilodalton macrocyclic peptides (with different scaffolds than natural peptides or small molecules) to a protein target with relatively low computational expense. Therefore, this study further validated and expanded the applicability of SuMD as a valuable protocol in studying varied molecular complex recognition.

The main advantages of the method used in this work are being able to correctly parameterize the ligand P2 of this class of macrocyclic peptides with a general Amber force field (GAFF) similar to small molecules and thus having no need for tailored parameterization due to the presence of unnatural amino acids and linkers, as well as the possibility to simulate the trajectory of a binding event in the nanosecond timescale thanks to SuMD. Consider that the association event starting from an unbound state is a rare event to be observed by cMD without the implementation of an enhanced sampling strategy. For instance, in Supplementary Video S4, a comparative cMD starting from the same state of SuMD is reported; during the 900 ns of simulation, P2 never approached thrombin, confirming the different sampling rate of the two methods. The opportunity of performing an efficient high-throughput molecular dynamics study of the remaining macrocyclic peptides of the same class, after further optimization and validation, can be envisioned. Therefore, the prospective use of this study’s findings would be toward using SuMD to perform high-throughput molecular dynamics studies of other available macrocyclic peptides of the same class, enjoying a highly diverse scaffold, to find probable hit candidates for various protein targets of interest and predict their binding mode as an adjunct predictive and screening tool, similarly to what was recently reported for fragments (Ferrari et al., 2021), narrowing down the requirement of going through experimental structural studies for each molecular complex of interest. On the contrary, a particular attention should be paid to the starting conformation of the macrocycle that could affect the recognition sampling since its flexibility could be rather pronounced. Specific methods (e.g., low-mode MD) are used to preprocess a novel ligand for selecting at least one of the few adequate starting conformations in solution. It should also be considered that a particularly flexible sub-kDa macrocycle could present more issue in sampling the bound conformation during the recognition. Anyway, all of these prospective enhancements would lead to the main goal of achieving computationally cheap molecular dynamics study methods with ever-increasing power in predicting experimentally equivalent final binding states and recognition of key elements and patterns of complexes.

## Data Availability

The original contributions presented in the study are included in the article/Supplementary Material, and further inquiries can be directed to the corresponding author.
