# Supplementary material for: Shedding Light on the Molecular Recognition of Sub-Kilodalton Macrocyclic Peptides on Thrombin by Supervised Molecular Dynamics
Source: Front Mol Biosci. 2021 Aug 31;8:707661. doi: 10.3389/fmolb.2021.707661 (PMC8438215; doi:10.3389/fmolb.2021.707661)
Supplement: Supplementary file 5 [file DataSheet1.docx]

Supplementary Material


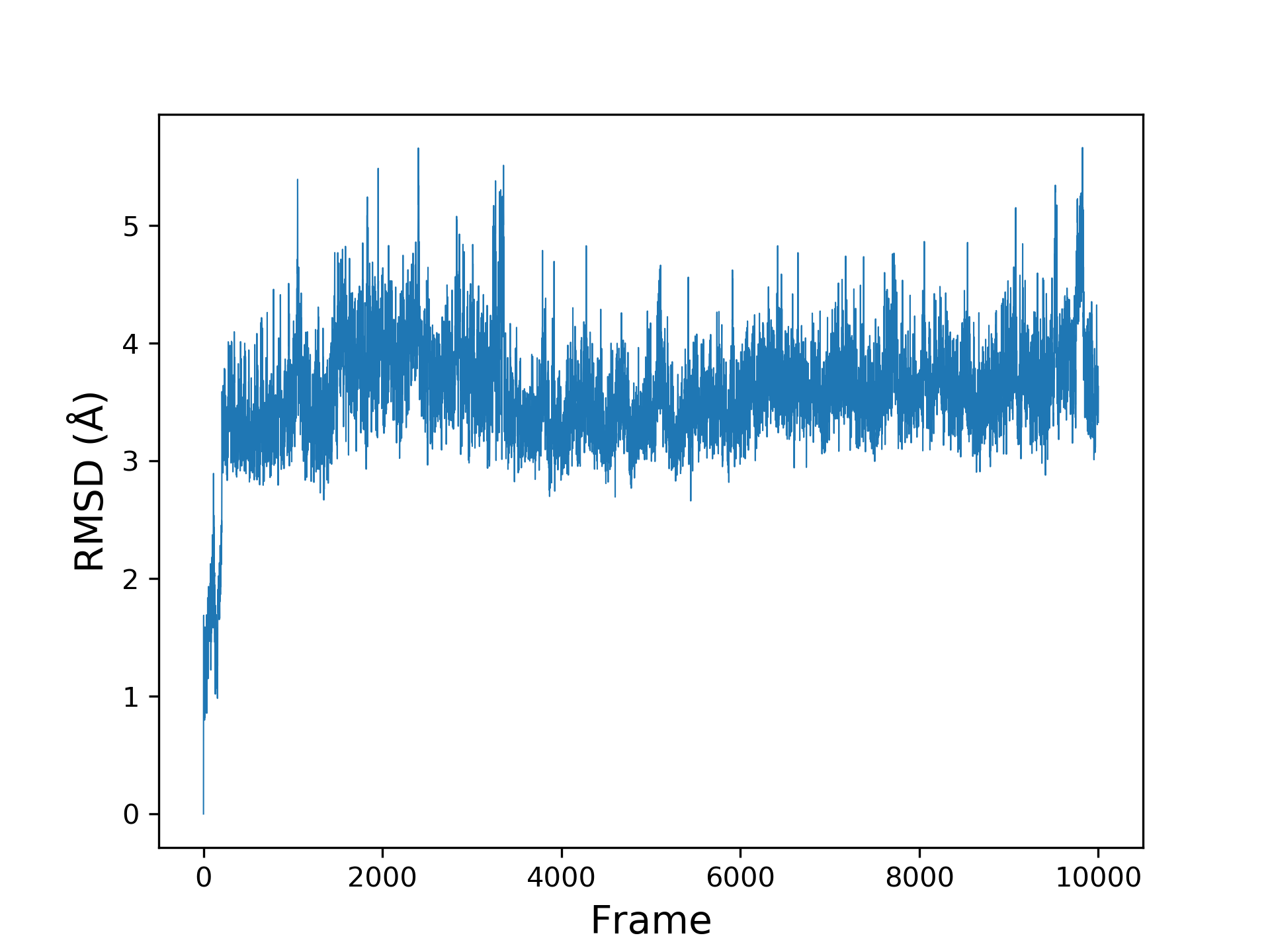


**Supplementary Figure 1**. RMSD plot of P2 during 200 ns free classical molecular dynamics (cMD) simulation started from X-ray final binding state (reference), equilibrated and relaxed in a fully explicit solvent environment.


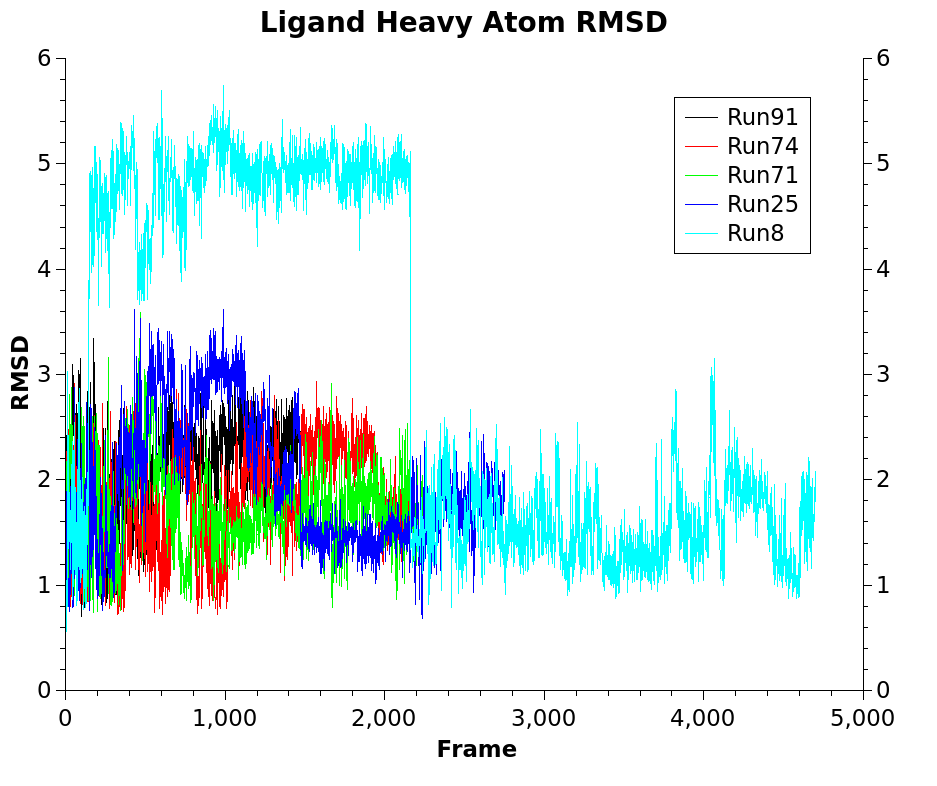


**Supplementary Figure 2**. RMSD plot of P2 in the best 5 replicas (Run: 8, 25, 71,74, 91) obtained by SuMD. The trajectories were superposed on the P2 heavy atom to highlight its flexibility during the recognitions.

**Captions of Supplementary Videos**

**Supplementary Video 1**. 200 ns of free classical molecular dynamics (cMD) simulation started from X-ray final binding state (reference), equilibrated and relaxed in a fully explicit solvent environment. During the initial 4 ns of the cMD a fluctuation within the range of experimental resolution (2.3 Å) was observed while, after 4 ns, a more significant shift of the macrocycle can be seen.

**Supplementary Video 2**. P2 SuMD replica 74 timeline representation; at the top left panel the produced trajectory from unbound to the bound state is shown; the top right panel shows the RMSD value of the ligand with respect to the reference during simulation time which also can indicate the distance between the ligand and active site centers of mass; the bottom left panel indicates the complex potential energy during the simulation time; and in the bottom right electrostatic interactions dynamic panel is seen, in which residue numbers refer to residue ID numbers assigned by VMD and the canonical numbering in thrombin respectively are: HIS57, TYR60A, PRO60C, TRP60D, TRP96, LEU99, GLU146, THR147, TRP147A, ASP170, SER171, THR172, ARG173, ILE174, ASP189, ALA190, CYS191, GLU192, VAL213, SER214, TRP215, GLY216, GLU217, GLY219, CYS220, ASP221A, ARG221, LYS224, GLY226, TYR 228.

**Supplementary Video 3**. The produced possible binding trajectory of P1 to thrombin (P1 SuMD replica 14 and classical molecular dynamics continuation for 50 ns) timeline representation; at the top left panel the produced trajectory from unbound to the bound state is shown; the top right panel shows the distance between the ligand and active site centers of mass during time; the bottom left panel indicates the complex potential energy during the simulation time; and in the bottom right electrostatic interactions dynamic panel is shown, in which residue numbers refer to residue ID numbers assigned by VMD and the canonical numbering in thrombin respectively are: HIS57, TYR60A, PRO60C, TRP60D, TRP96, ARG 97, LEU99, GLU146, THR147, TRP147A, THR172, ARG173, ILE174, ASP189, ALA190, CYS191, GLU192, SER195, VAL213, SER214, TRP215, GLY216, GLU217, GLY219, CYS220, ASP221A, TYR225, GLY226, PHE227, TYR 228.

**Supplementary Video 4.** Classical unsupervised molecular dynamics simulation of P2 and thrombin. The starting state of the system was the same as P2 SuMD replicas. During a long simulation time (870 ns), no contact or insightful binding event can be observed, although with SuMD in less than 100 ns we could obtain full binding trajectories. This video can reflect the efficiency of the supervision protocol in sampling binding events and production of binding trajectories.
